# Reduction in COVID-19 Vaccine Effectiveness against SARS-CoV-2 Variants in Seoul according to Age, Sex, and Symptoms: A Test-Negative Case-Control Study

**DOI:** 10.3390/ijerph192416958

**Published:** 2022-12-16

**Authors:** Hyerin Gim, Soyoung Oh, Heeda Lee, Seul Lee, Haesook Seo, Yumi Park, Jae-Hyun Park

**Affiliations:** 1 Infectious Disease Research Center, Citizen’s Health Bureau, Seoul Metropolitan Government, 110, Sejong-daero, Jung-gu, Seoul 04524, Republic of Korea; 2 Citizen’s Health Bureau, Seoul Metropolitan Government, 110, Sejong-daero, Jung-gu, Seoul 04524, Republic of Korea; 3 Department of Social and Preventive Medicine, Sungkyunkwan University School of Medicine, 2066 Seobu-ro, Jangan-gu, Suwon 16419, Republic of Korea

**Keywords:** COVID-19, mRNA vaccines, adenovirus vaccines, vaccine efficiency, risk factors

## Abstract

Background: We evaluated vaccine effectiveness (VE) against infections with SARS-CoV-2 variants of concern in Seoul, the capital of the Republic of Korea, having the highest population density in the country, under real-world conditions. Methods: We evaluated the reduction in the effectiveness of mRNA and viral-vector COVID-19 vaccines against infection by the SARS-CoV-2 delta variant in a subpopulation from April 2021 to July 2021 who visited screening clinics in Seoul using a test-negative case-control study design. Moreover, we conducted a case-control study matching the ten-year-old age group, sex, healthcare workers, and five districts of Seoul, which are considered confounding factors. Results: The full VE in the pre-delta-dominant period was 95.0% (95% confidence interval [CI]: 91.2–97.2); however, it decreased to 61.1% (95% CI: 53.2–67.6) during the delta-dominant period. Notably, we found that COVID-19 VE was significantly decreased in individuals aged ≥80 years (52.9%, 95% CI: −9.9–79.8), men (50.6 %, 95% CI: 39.4–59.8), and asymptomatic individuals (49.8%, 95% CI: 36.5–60.3) during the widespread SARS-CoV-2 delta variant circulation. Conclusions: Vaccine-mediated protection drastically declined during the delta-dominant period and in vulnerable groups. This study suggests the requirement for additional countermeasures, such as the administration of a booster vaccine, in vulnerable groups based on age, sex, and symptomatic manifestation.

## 1. Introduction

The outbreak of severe acute respiratory syndrome coronavirus 2 (SARS-CoV-2) has resulted in the coronavirus disease 2019 (COVID-19) pandemic. Several vaccines have been developed against COVID-19 and are now commercially available. In a randomized placebo-controlled phase III trial, the BNT162b2 mRNA (Pfizer-BioNTech) and the mRNA-1273 (Moderna) vaccines had 94–95% efficacy against SARS-CoV-2 after the second dose [1,2,3]. The ChAdOx1 (AstraZeneca) and Ad26.COV2.S adenoviral vector (Janssen/Johnson & Johnson) vaccines presented 70 and 66.9% efficacy against SARS-CoV-2 after the second and first inoculations, respectively [4,5]. Moreover, COVID-19 vaccines can prevent SARS-CoV-2 infections in a real-world scenario [6,7,8].

However, as of January 2022, the World Health Organization (WHO) designated five SARS-CoV-2 variants of concern (VOCs): alpha, beta, gamma, delta, and omicron. Owing to mutations in the spike protein, SARS-CoV-2 VOCs show decreased susceptibility to antibody neutralization and increased transmissibility [9,10,11,12,13], which contribute to their global spread. Therefore, as new SARS-CoV-2 VOCs dominate in many countries worldwide, ongoing re-evaluations of COVID-19 vaccine effectiveness (VE) are urgently needed [14,15,16]. Concerns have been raised regarding decreased VE caused by the waning of vaccine protection over time [17,18,19,20], individual differences in immune responses, and adherence to social distancing. In a randomized trial, a decrease in VE due to the appearance of variants has been demonstrated [21,22]; however, the reduction in VE in the real world may show a different pattern. In the real world, VE is affected by sex, age, individual immunity, and the degree of adherence to social distancing; therefore, these factors should be considered when assessing VE.

Furthermore, as viral infections are dependent on population densities [23], evaluations of VE in nations with high population densities are required. However, there are few studies on COVID-19 VE against SARS-CoV-2 VOC infection in countries with high population densities. As of 2018, in Our World in Data, the population density of the Republic of Korea has been included in the top 10% worldwide. Moreover, in the real world, VE evaluation methods include cohort studies and case-control studies [24]. However, cohort studies are limited by the need for large sample sizes and high costs, whereas case-control studies may give rise to bias, which can influence the outcome. The test-negative case-control study design (TND) is a powerful method for evaluating VE against influenza and other respiratory viruses, as it can minimize biases by collecting cases and controls from the same communities [25,26]. However, there are few studies on VE against infections by SARS-CoV-2 VOCs in sub-populations. We conducted a TND study by collecting the COVID-19 test results of people visiting the designated/temporary screening clinics.

This study aimed to evaluate VE against infections by SARS-CoV-2 VOCs in Seoul, the capital of the Republic of Korea, having the highest population density in the country, under real-world conditions using the TND method.

## 2. Materials and Methods

### 2.1. Study Design

We used the TND method to estimate VE against SARS-CoV-2 infection. In Seoul, there are designated/temporary screening clinics that test for COVID-19. These systems are operated to allow citizens to freely receive COVID-19 tests regardless of their symptoms. Moreover, there are designated screening clinics for testing individuals who had been in close contact with patients with COVID-19. In this study, we considered individuals who visited screening clinics to confirm a COVID-19 infection as the population in the same community, thereby reducing bias.

**Figure 1:**
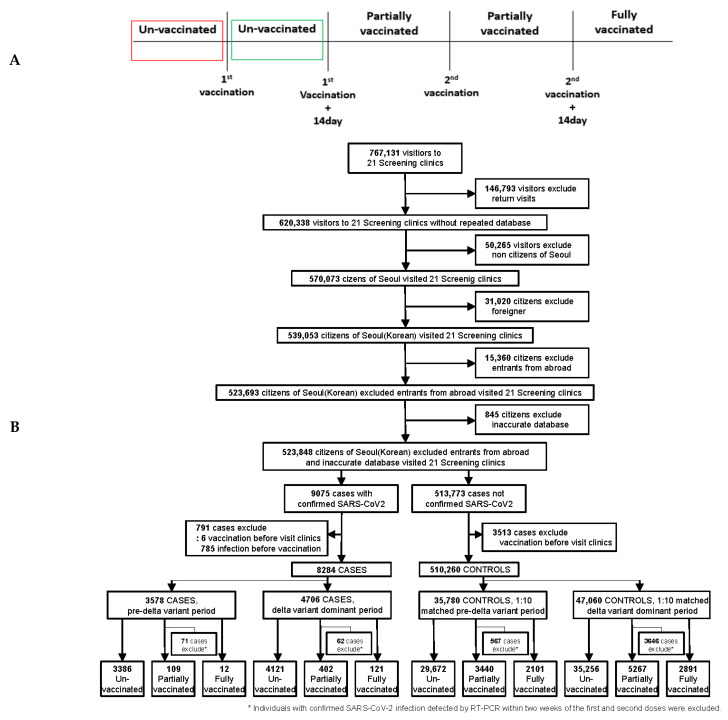
Classification criteria for the vaccination groups and flowchart of cases and controls selected in the pre-delta-dominant period and the delta-dominant period. (**A**) Classification criteria for the vaccination group established in consideration of vaccination and whether 14 days had elapsed from the date of vaccination. (**B**) Flowcharts showing the selection process for cases (PCR-positive) and controls (PCR-negative) in the pre-delta-dominant period and the delta-dominant period to investigate the reduction in COVID-19 vaccine effectiveness against SARS-CoV-2.

Antibody titers are highest and neutralization reaction levels on the 14th day after a single SARS-CoV-2 inoculation [27]; therefore, the 14th day was used as the reference date for full antibody production [28,29]. In those who received only the first dose of COVID-19 vaccination, individuals in which 14 days had passed after their first COVID-19 vaccination were classified as partially immunized. In those who received their second dose of COVID-19 vaccination (excluding those whose first dose was the Ad26.COV2.S adenoviral vector vaccine), individuals in which 14 days had not passed after their second COVID-19 vaccination were classified as partially immunized. Individuals whose 14 days had passed after their second COVID-19 vaccination were classified as fully immunized (Figure 1A).

In this study, the rates of effectiveness of partial and complete vaccinations during the study period (from April 2021 to July 2021) were 49.9 and 79.7%, respectively, lower than those reported in previous studies, and were estimated to stem from the spread of the delta variant (Table S4) [28]. Accordingly, based on the results in July, when the delta variant rapidly spread, the control groups were extracted ten-fold according to the cases identified from April–June (*n* = 3578) and July (*n* = 4706), respectively, considering confounding factors (Figure 1B). In the April–June, and July groups, after the first dose of the COVID-19 vaccine, those infected before full antibody production (*n* = 71, *n* = 62) and those showing non-full antibody levels (*n* = 567, *n* = 3646) were classified as the unvaccinated group (Figure 1B).

In this study, we followed the checklist in the Strengthening of the Reporting of Observational Studies in Epidemiology (STROBE) guidelines for case-control studies (Table S1).

### 2.2. Study Population

The selection criteria were adults aged >19 years who lived in Seoul with complete information in data sources on sex, age, district of Seoul, vaccination data, and real-time reverse transcription polymerase chain reaction (RT-PCR) testing data. All SARS-CoV-2 infections were confirmed by RT-PCR. Individuals with confirmed SARS-CoV-2 infection detected by RT-PCR within two weeks of the first and second doses were excluded. Case (PCR-positive) and control (PCR-negative) groups were divided according to their RT-PCR test results from nasopharyngeal swabs. Symptomatic infections were defined as PCR-positive results with symptoms such as a cough, sputum, soreness, chills, headache, or fever etc. known to be a symptom of COVID-19 infection in the WHO definition. Moreover, asymptomatic infections were defined as PCR-positive without any of the aforementioned clinical signs.

### 2.3. Data Sources

In this study, screening of clinic visitors, vaccination inoculation, and the SARS-CoV-2 infection database (DB) were used as data sources. The study group consisted of those who visited screening clinics to confirm COVID-19 infection, as registered in the system of the Korea Centers for Disease Control and Prevention. The Seoul Metropolitan Government has constructed the SARS-CoV-2 infection DB, and the use of this DB was permitted as the data were reported from all screening clinics in Seoul. The DB includes infection numbers, individual names, RT-PCR cycle threshold values, resident registration numbers, sex, symptoms, hospital admissions, incidences of death, jobs, and other information.

To establish the vaccine DB, data on individuals who were vaccinated between 26 February 2021 and 31 July 2021 were extracted from the system of Korea Centers for Disease Control and Prevention. The vaccination DB includes the name of the individual, resident registration number, cell phone number, vaccination number, vaccine manufacturer, and inoculation date. The date of birth (dd/mm/yyyy), name, and residential addresses from the three DBs mentioned above were considered as linked key data and were extracted. Therefore, the individual vaccination and infection DB were merged for individuals in the screening clinic DB using a linked key.

A matched case-control study could control for matching factors that were considered confounding factors [30], which included the ten-year-old age group, sex, healthcare workers, and five districts of Seoul. Healthcare workers included doctors, dentists, oriental medicine doctors, veterinarians, nurses, nursing assistants, pharmacists, herbal medicine pharmacists, medical technicians, and physical therapists in accordance with the Korea Employment Classification of Occupation. Additionally, the five districts of Seoul comprised 25 autonomous districts.

### 2.4. Statistical Analysis

A chi-square test [31,32] was used to estimate the odds ratio with 95% confidence intervals (CI) using the Statistical Package for Social Sciences (SPSS) statistical software package (IBM Corp. Released 2016, IBM SPSS Statistics for Windows, Version 24.0., IBM Corp., Armonk, NY, USA).

Before selecting 1:10 data samples, we conducted a pairwise two-sample *t*-test based on the null hypothesis, H0, which holds that the means of the two populations (case and control) are equal due to confounders. Data samples were selected by 1:10 frequency matching by confounders. There were no significant differences (*p* = 1) between the two groups based on confounders. Therefore, after 1:10 frequency matching, there were no differences in confounders between cases and controls.

Analyses were conducted to estimate VE against SARS-CoV-2 infection. Since the odds ratio (OR) was calculated by matching cases and controls, the derived OR was adjusted. The OR was calculated by comparing the odds of vaccination among RT-PCR-positive cases to those of RT-PCR-negative controls. VE was calculated as (1 − OR) × 100 (%) [33,34]. We used the following formulae to calculate the odds ratios (OR) and its confidence intervals (CI). OR = a × d/b × c, where: a is the number of times both A and B are present, b is the number of times A is present, but B is absent, c is the number of times A is absent, but B is present, and d is the number of times both A and B are negative.

$$\mathrm{VE}=1-\frac{vaccinated\;among\;cases\times unvaccinated\;among\;controls}{vaccinated\;among\;controls\times unvaccinated\;among\;cases\;}$$

To calculate the confidence intervals, we use the log odds ratio, log(OR) = log(a × d/b × c), and calculate its standard error(SE):se(log(or)) = √1/a + 1/b + 1/c +1/d. The confidence interval is calculated as: = exp(log(or) ± Zα/2 × √1/a + 1/b + 1/c + 1/d). We selected cases (*n* = 8151) and controls (*n* = 78,627), which are number of people in the population exceeding the minimum sample size, to maintain 80% power.

## 3. Results

### 3.1. Participants

Between 1 April 2021 to 31 July 2021, 767,131 visitors of 21 screening clinics for SARS-CoV-2 testing were included in the cohort group. Among these, repeated visitors (*n* = 146,793), non-residents of Seoul (*n* = 50,265), foreigners (*n* = 31,020), entrants from abroad (*n* = 15,360), and inaccurate vaccine database entries (*n* = 845) were excluded. Moreover, those with a history of COVID-19 vaccination before visiting the screening clinics were excluded from the case (*n* = 6) and control (*n* = 3513) groups (Figure 1B). Moreover, individuals infected before vaccination were excluded (*n* = 785). In the remaining pre-matching study group (*n* = 518,544), the ratio of cases (*n* = 8284) to controls (*n* = 510,260) was 1:62 (Table S2), which was adjusted to ensure accuracy in the study.

### 3.2. Vaccine Coverage

**Figure 2:**
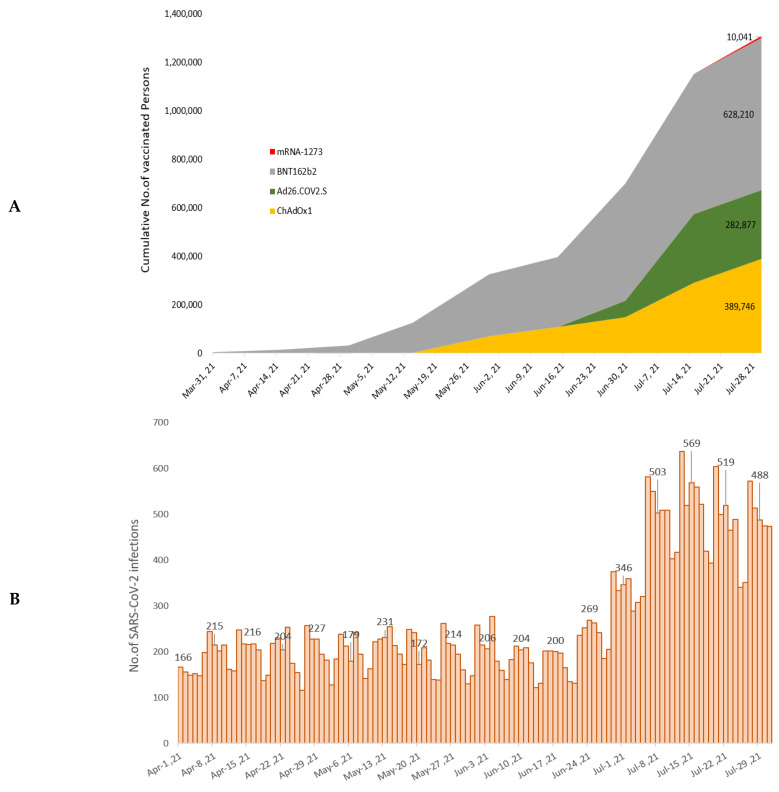

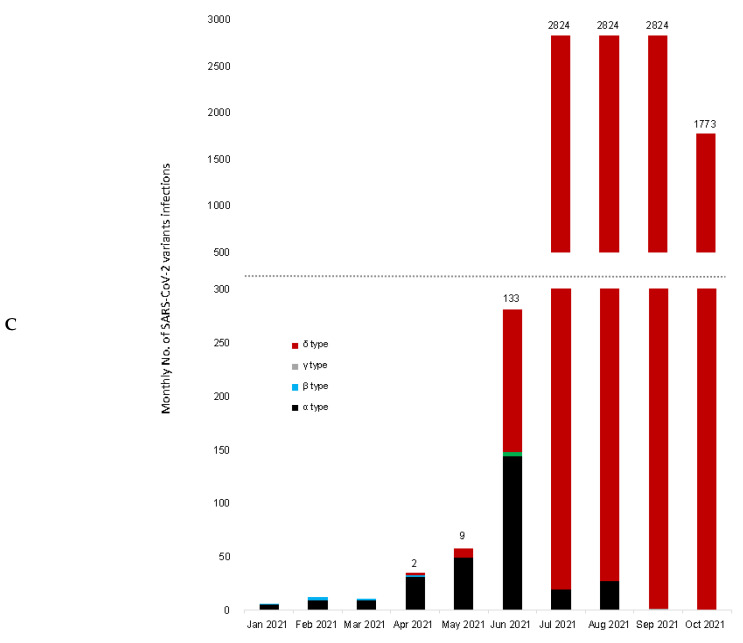
Cumulative number of COVID-19-vaccinated individuals, the incidence of daily SARS-CoV-2 infections, and the number of monthly SARS-CoV-2 variant infections in Seoul between April 2021 and July 2021. (**A**) Number of weekly cumulative COVID-19 vaccinated individuals as confirmed by the Daily Report of the Seoul Metropolitan Government. (**B**) Incidence of daily SARS-CoV-2 infections based on RT-PCR test results (positive). (**C**) Number of monthly SARS-CoV-2 variant infections based on the results of whole genome sequencing or partial spike gene sequencing.

By the end of the study period (31 July 2021), approximately 13.34% of the Seoul population (1,310,874/9,828,094) had been fully vaccinated and approximately 35.78% (3,516,768/9,828,094) had been partially vaccinated. From April to July 2021, 628,210, 389,746, 282,877, and 10,041 individuals had received the BNT162b2, ChAdOx1, Ad26.COV2.S, and mRNA-1273 COVID-19 vaccines, respectively (Figure 2A). However, the results of an analysis based on visitors of screening clinics showed that the percentage of those vaccinated in the population tended to decrease. The complete vaccination rate of the pre-matching study population was 7.0% (36,097/518,544) (Table S2), whereas that of the post-matching study population was 6.5% (5934/91,124) (Table S3). Moreover, in the separate analyses of April–June and July, the vaccination coverage rates were 5.4% (2113/39,358) in April–June and 5.8% (3012/51,766) in July (Table 1).

### 3.3. Study Population

Figure 1B presents a flowchart of the selection process of cases (PCR-positive) and controls (PCR-negative) from April–June and July during the VE evaluation. Table 1 lists the demographic and regional characteristics, the presence or absence of symptoms, PCR test results, and the vaccination status of the study groups. We used a 10-year age band as a matching factor, and the mean ages of confirmed cases in April–June and July were 39.2 (SD:16.8) and 36.8 years (SD:17.1), respectively. The case group in July showed higher proportions in the 0–9, 10–19, 20–29, and 60–69 age groups compared to that in April–June. Additionally, the case group in July had a higher ratio of non-healthcare workers and symptomatic infections compared to that in April–June. However, there was no significant difference in the sex ratio between the two periods (Table 1). In both April–June and July, among the five districts of Seoul, the northeast, southeast, and southwest areas accounted for a high proportion of the cases (Table 1), with Seongdong, Nowon, Gangnam, Guangdong, Dongjak, and Gwanak significantly contributing to these proportions (Table S6).

### 3.4. COVID-19 VE against SARS-CoV-2 Infections

In April–June, 12 fully vaccinated and 109 partially vaccinated patients were confirmed as SARS-CoV-2-infected patients. In July, 121 fully vaccinated and 402 partially vaccinated individuals were confirmed as infected (Table S5). In other words, among the infected, the proportion of vaccinated individuals increased from 3.3% in April–June (3.0% partial vaccination, 0.3% complete vaccination) to 11.1% in July (8.5% partial vaccination, 2.6% complete vaccination). From April to June, VE increased with complete vaccination (95.0%, 95% CI: 91.2–97.2) compared to partial vaccination (72.3%, 95% CI: 66.4–77.2) (Table 2). However, both partial and complete VE decreased to 29.0% (95% CI: 21.1–36.2) and 61.1% (95% CI: 53.2–67.6) in July, respectively.

### 3.5. COVID-19 VE against SARS-CoV-2 Infection according to Age and Sex

**Figure 3:**
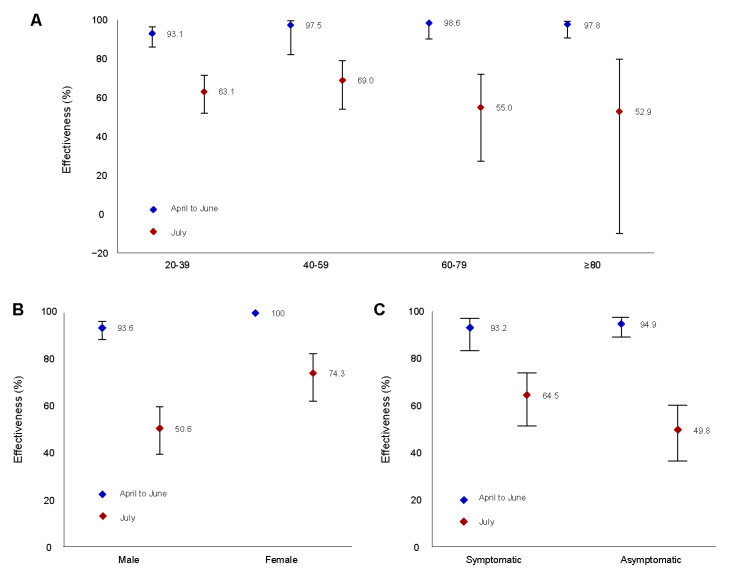
Effectiveness of COVID-19 vaccines against SARS-CoV-2 infection according to age, sex, and symptoms. Estimation of vaccine effectiveness in the pre-delta-dominant period (blue diamonds) and the delta-dominant period (red diamonds) and 95% confidence intervals (vertical bars) according to (**A**) age, (**B**) sex, and (**C**) symptoms. The age groups were divided into 20-year intervals.

We evaluated VE according to age and sex in these subgroups (Table 2). VE for the age subgroup was analyzed by dividing age groups at 20-year intervals. From April to June, the effectiveness of complete vaccination tended to increase among older adults. However, in July, the effectiveness of complete vaccination in the 60–79 age group (55.0%, 95% CI: 27.4–72.2) and the 80+ age group (52.9%, 95% CI: −9.9–79.8) was lower than that in the 20–39 (63.1%, 95% CI: 52.0–71.7) and 40–59 age groups (69.0%, 95% CI: 54.0–79.1). Moreover, in both April–June and July, the effectiveness of partial vaccination for the 80+ age group was negligible. VE was higher in individuals that were completely vaccinated than in those who were partially vaccinated, regardless of sex. Based on sex stratification, the VE for women was 93.6 (95% CI: 88.7–96.4) and 74.3% (95% CI: 62.2–82.5), and that for men was 100 and 50.6% (95% CI: 39.4–59.8) in April–June and July, respectively (Figure 3A,B).

### 3.6. COVID-19 VE against Symptomatic and Asymptomatic Infections

The COVID-19 VE against symptomatic and asymptomatic infections was higher in the fully vaccinated group than in the partially vaccinated group, regardless of the presence or absence of symptoms, which is consistent with the findings of overall effectiveness (Table 2). Based on the presence or absence of symptoms, the VE against symptomatic infections was higher than that against asymptomatic infections in the partially vaccinated group in April–June (Table 2). From April to June, the VE was 94.9% (95% CI: 89.2–97.6) against asymptomatic infections and 93.2% (95% CI: 83.5–97.2) against symptomatic infections whereas in July, the VE was 49.8% (95% CI: 36.5–60.3) against asymptomatic infections and 64.5% (95% CI: 51.3–74.0) against asymptomatic infections (Figure 3C).

## 4. Discussion

Comparative analyses of COVID-19 VE in certain subgroups have rarely been conducted. In the present study, we conducted a comparative analysis of the COVID-19 VE involving three subgroups (age, sex, symptoms) using the TND method, which was adjusted for confounding factors. We revealed that the VE in the real world decreased over time, which is consistent with the findings of earlier studies. Moreover, the VE was noticeably lower in July than in April–June, likely due to several factors. First, spike mutation detection tests in SARS-CoV-2-positive individuals revealed that the proportion of individuals with the delta variant, which spreads faster than other strains, surged rapidly in July. Second, the waning of the vaccine was affected by differences in personal immunity and compliance with social distancing, which may have affected the VE. Additionally, individuals in the older adults, male, and asymptomatic groups showed greater reductions in VE than those in other comparative subgroups.

### 4.1. Social Distancing

In this study, we measured the extent of VE reduction by comparing cases in April–June to those in July, when the delta variant became dominant. Moreover, influential risk factors were inferred and groups vulnerable to breakthrough infections were identified through a comparative analysis of VE within subgroups.

According to a previous study in the US, within two weeks of the implementation of the national stay-at-home order, the adherence to social distancing evaluated using objective indicators increased to approximately 35%. Based on this study, the Korean government strengthened social distancing on 12 July 2021. Therefore, our results indicate that the effect of strengthening the social distancing policy for VE during the study period was poor. However, it is necessary to estimate the adherence of each individual to the social distancing policy more closely because the lower the degree of adherence to the social distancing policy, the higher the risk of exposure to infection, which may reduce VE.

### 4.2. Age

In the present study, there were no significant differences in VE for each age group from April to June; however, in July, the VE remarkably decreased in older adults and the standard deviation by age group increased. Since the vaccination schedule for each age group was different, individuals over 80 years of age completed their vaccination approximately one to two months earlier than other age groups. The natural waning of vaccine protection may show up to a 10% decrease between one and two months after vaccination [17,18,19,20]. However, there are additional factors other than the waning effect that can cause a large decrease in VE. As the VE decreased by an average of 30–45% after a rapid increase in the infection rate with the delta variant in all age groups, the effect of the delta variant on the VE cannot be ignored. Furthermore, aging of the immune system [35] and the presence of various underlying diseases in individuals [22,23,24] may have also contributed to the decline in VE. As the delta variant began to predominate, the effects of these individual characteristics were amplified with age, increasing the gap in the standard deviation among age groups. In other words, even if the waning effect and individual immunity are considered, when the delta variant, which spreads more rapidly than other strains, surges, the VE becomes weaker and more diversified according to individual characteristics as the age increases.

### 4.3. Sex

Previous findings showing differences in VE according to sex are limited; therefore, we conducted a subgroup analysis with frequency matching for a case-control group to increase the reliability of our result compared to those in previous research. In our study, based on sex stratification, the VE was higher in women than in men during the study period, consistent with a previous study [36,37]. The degree of difference in the VE between men and women was more pronounced in July, and the difference in the degree of the decrease in the VE between men and women was 17% and was more pronounced in men. The overall decrease in VE is likely due to generational changes in the dominant viral species. Furthermore, differences in compliance with social distancing due to differences in community lifestyles according to sex may also have had an effect. Based on the ratio of social activities of men to those of women in the study population, 72% of men and 57% of women were economically active from April to June, showing a difference of approximately 15%, and all breakthrough infections were in men. In July, 67% of men and 42% of women were economically active, showing a difference of approximately 25%, and the number of breakthrough infections was four times higher in men than in women. According to national monitoring indicators, men generally have a higher economic activity participation rate than women [38], and our study population showed a similar rate.

In summary, men were more socially active than women; as the gap in economic activity between sexes increased, the number of breakthrough infections increased. We analyzed the sex-based VE adjusted for five types of confounders and revealed that the presence or absence of social activity, an external factor, may have affected the VE. Moreover, the VE is expected to be affected by differences in biological characteristics [39,40] and hygiene management behavior [41,42]. Our results suggest that the reduction in VE was greater in men than in women when rapidly spreading variants were predominant. However, further studies are required to support these results, as detailed studies on these findings are scarce.

### 4.4. Symptoms

Previous studies have shown that the COVID-19 VE against symptomatic infections was slightly greater than that against asymptomatic infections [43,44]. However, in our study, the VE for asymptomatic infections was comparable to that for symptomatic infections. The VE in July compared with April–June decreased to 45.1% (49.8% vs. 94.9%) for asymptomatic infections and 28.7% (64.5% vs. 93.2%) for symptomatic infections, indicating that those with asymptomatic infections experienced a greater decrease in VE than those with symptomatic infections. From April to June, the differences in the effectiveness of partial vaccination and complete vaccination according to the presence or absence of symptoms were 2.0% (70.0% vs. 68.0%) and 1.7% (93.2% vs. 94.9%), respectively. However, in July, the corresponding differences in the effectiveness of partial vaccination and complete vaccination according to the presence or absence of symptoms were greater at 11.7% (20.6% vs. 8.9%) and 14.7% (64.5% vs. 49.8%), respectively. Moreover, during July, when the proportion of the delta variant exceeded 50%, the VE for symptomatic infections was greater than that for asymptomatic infections, which is consistent with a previous report [7,45]. This result indicates that even when considering the maximum waning effect of 10% that can occur over three months [11,13,14,15], the VE decreased more in individuals with asymptomatic infections than those with symptomatic infections as the proportion of rapidly spreading variants increased. This result may be attributed to a lack of adherence to social distancing by asymptomatic individuals because of the absence of symptoms compared to symptomatic individuals. In a previous study in which individual respiratory infections were rife, and the social distancing policy was identically applied regardless of the presence or absence of symptoms, the infection suppression effect was greater in the asymptomatic group than that in the symptomatic group [46]. Therefore, encouraging asymptomatic individuals to adhere to social distancing may increase the VE. Nevertheless, detailed studies of the epidemiological and biological basis for symptomatic or asymptomatic infections are required to further clarify these observations.

### 4.5. Limitations

Our study has several limitations. The study population showed a slightly different proportion compared to the age group composition of Seoul; therefore, the results cannot be generalized. Moreover, observational studies may be unexpectedly biased and should be interpreted with caution. The differences in subgroup results compared to those in other studies can be explained by various factors, such as certain characteristics, time of vaccination, and the predominant variant. Although we observed a significant decrease in the VE during the delta variant-dominant period in the older age group, it was difficult to interpret this result as being entirely due to the delta variant risk, as the vaccination date was not adjusted. Finally, we did not adjust for the difference in the VE based on the type of vaccine, despite all four licensed vaccines being used in Seoul.

This study minimized bias with frequency matching in the real world and identified a special population group vulnerable to infection, even after vaccination, through subgroup analysis, which has not been previously attempted. This study suggests that vaccination policies should focus on certain vulnerable groups to increase the VE and reduce the spread of COVID-19. The effective control of COVID-19 infections is possible by strengthening social distancing policies, boosting shots, and shortening the vaccination period, especially for people over 60 years of age, men, and those who are asymptomatic. Therefore, as new SARS-CoV-2 VOCs appear, it is necessary to either increase the vaccination rates or promote booster shots in vulnerable groups.

## 5. Conclusions

In this study using DBs including screening clinic visitors, vaccination, and the SARS-CoV-2 infection, effectiveness of COVID-19 vaccine showed a decline during a delta-variant dominant period. We found that those over 80 years of age, males, and asymptomatic groups were more susceptible than people of other ages, females, and those who were symptomatic during widespread the SARS-CoV-2 delta variant circulation. Evidence from this study supports the conclusion that the vulnerable groups in policies, which should strive to increase VE and reduce the spread of COVID-19.

## Figures and Tables

**Table 1:** Comparison of the characteristics of the study population according to SARS-CoV-2 infection (detected by RT-PCR) and COVID-19 vaccination statuses.

Characteristics	SARS-CoV-2 Infection								Vaccination											
	April to June				July				April to June						July					
	Controls		Cases		Controls		Cases		Unvaccinated		Partially vaccinated		Fully vaccinated		Unvaccinated		Partially vaccinated		Fully vaccinated	
Total population	35,780		3578		47,060		4706		33,696		3549		2113		43,085		5669		3012	
Age, mean (SD), years	39.3	(16.8)	39.2	(16.8)	36.9	(16.9)	36.8	(17.1)	39.3	(16.8)	39.7	(16.6)	39.9	(16.7)	36.8	(17.0)	37.9	(16.4)	38.2	(16.3)
Age group, *n* (%), years																				
0–9	1260	(3.5)	126	(3.5)	2360	(5.0)	236	(5.0)	1386	(4.1)	0	(0.0)	0	(0.0)	2596	(6.0)	0	(0.0)	0	(0.0)
10–19	2190	(6.1)	219	(6.1)	4640	(9.9)	464	(9.9)	2409	(7.1)	0	(0.0)	0	(0.0)	5099	(11.8)	4	(0.1)	1	(0.0)
20–29	7440	(20.8)	744	(20.8)	11,520	(24.5)	1152	(24.5)	8083	(24.0)	59	(1.7)	42	(2.0)	11,999	(27.8)	322	(5.7)	351	(11.7)
30–39	8410	(23.5)	841	(23.5)	8450	(18.0)	845	(18.0)	7872	(23.4)	346	(9.7)	1033	(48.9)	7666	(17.8)	443	(7.8)	1186	(39.4)
40–49	6560	(18.3)	656	(18.3)	8090	(17.2)	809	(17.2)	6487	(19.3)	459	(12.9)	270	(12.8)	7552	(17.5)	867	(15.3)	480	(15.9)
50–59	5750	(16.1)	575	(16.1)	7310	(15.5)	731	(15.5)	5705	(16.9)	522	(14.7)	98	(4.6)	6927	(16.1)	809	(14.3)	305	(10.1)
60–69	2450	(6.8)	245	(6.8)	3330	(7.1)	333	(7.1)	1181	(3.5)	1476	(41.6)	38	(1.8)	960	(2.2)	2558	(45.1)	145	(4.8)
70–79	1190	(3.4)	119	(3.4)	1080	(2.2)	108	(2.2)	402	(1.1)	650	(18.3)	257	(12.2)	211	(0.5)	655	(11.5)	322	(10.8)
80–89	440	(1.2)	44	(1.2)	240	(0.5)	24	(0.5)	118	(0.4)	34	(1.0)	332	(15.7)	54	(0.2)	10	(0.2)	200	(6.6)
≥90	90	(0.3)	9	(0.3)	40	(0.1)	4	(0.1)	53	(0.2)	3	(0.1)	43	(2.0)	21	(0.1)	1	(0.0)	22	(0.7)
Sex, *n* (%)																				
Male	17,940	(50.1)	1794	(50.1)	23,770	(50.5)	2377	(50.5)	16,396	(48.7)	1715	(48.3)	1623	(76.8)	21,643	(50.2)	2492	(44.0)	2012	(50.5)
Female	17,840	(49.9)	1784	(49.9)	23,290	(49.5)	2329	(49.5)	17,300	(51.3)	1834	(51.7)	490	(23.2)	21,442	(49.8)	3177	(56.0)	1000	(49.5)
Symptom, *n* (%)																				
Symptomatic	4365	(12.2)	1631	(45.6)	6006	(12.8)	2435	(51.7)	5464	(16.2)	347	(9.8)	185	(8.8)	7505	(17.4)	586	(10.3)	350	(11.6)
Asymptomatic	31,415	(87.8)	1947	(54.4)	41,054	(87.2)	2271	(48.3)	28,232	(83.8)	3202	(90.2)	1928	(91.2)	35,580	(82.6)	5083	(89.7)	2662	(88.4)
Health care workers, *n* (%)																				
Health care	140	(0.4)	14	(0.4)	111	(0.2)	10	(0.2)	85	(0.3)	63	(1.8)	6	(0.3)	66	(0.2)	20	(0.4)	35	(1.2)
Non-health care	35,640	(99.6)	3564	(99.6)	46,949	(99.8)	4696	(99.8)	33,611	(99.7)	3486	(98.2)	2107	(99.7)	43,019	(99.8)	5649	(99.6)	2977	(98.8)
Districts of Seoul, *n* (%)																				
Center Area	910	(2.5)	91	(2.5)	1440	(3.1)	144	(3.1)	849	(2.5)	116	(3.3)	36	(1.7)	1309	(3.0)	186	(3.3)	89	(3.0)
Northeast Area	8540	(23.9)	854	(23.9)	10,610	(22.5)	1061	(22.5)	7895	(23.4)	944	(26.6)	555	(26.3)	9644	(22.4)	1298	(22.9)	729	(24.1)
Northwest Area	640	(1.8)	64	(1.8)	3380	(7.2)	338	(7.2)	604	(1.8)	62	(1.7)	38	(1.8)	3209	(7.4)	347	(6.1)	162	(5.4)
Southeast Area	18,210	(50.9)	1821	(50.9)	15,900	(33.8)	1590	(33.8)	17,380	(51.6)	1674	(47.2)	977	(46.2)	14,941	(34.7)	1606	(28.3)	943	(31.3)
Southwest Area	7480	(20.9)	748	(20.9)	15,730	(33.4)	1573	(33.4)	6968	(20.7)	753	(21.2)	507	(24.0)	13,982	(32.5)	2232	(39.4)	1089	(36.2)

Cases and controls were 1:10 frequency matched by age group, sex, healthcare workers, and five districts of Seoul; Continuous variables are denoted as mean (SD); categorical variables are denoted as *n* (%); Healthcare workers are defined by the Korea Employment Classification of Occupation (KECO); Seoul is divided into five districts, referred to here as the central, northeast, northwest, southeast, and southwest areas.

**Table 2:** Reduction in COVID-19 vaccine effectiveness against SARS-CoV-2 infection according to age, sex, and symptoms.

	April to June Effectiveness, % (OR Value)				July Effectiveness, % (OR Value)			
	Partially vaccinated		Fully vaccinated		Partially vaccinated		Fully vaccinated	
**Age group, years (%)**	VE (95% CI)	Odds ratio (95% CI)	VE (95% CI)	Odds ratio (95% CI)	VE (95% CI)	Odds ratio (95% CI)	VE (95% CI)	Odds ratio (95% CI)
20–39	49.4 (21.2; 67.5)	0.506 (0.325; 0.788)	93.1 (86.1; 96.5)	0.069 (0.035; 0.139)	80.3 (67.6; 88.0)	0.197 (0.120; 0.324)	63.1 (52.0; 71.7)	0.369 (0.283; 0.480)
40–59	78.1 (66.8; 85.6)	0.219 (0.144; 0.332)	97.5 (82.3; 99.7)	0.025 (0.003; 0.177)	58.2 (46.9; 67.1)	0.418 (0.329; 0.531)	69.0 (54.0; 79.1)	0.310 (0.209; 0.460)
60–79	90.7 (87.3; 93.2)	0.093 (0.068; 0.127)	98.6 (90.2; 99.8)	0.014 (0.002; 0.098)	−1.6 (−27.6; 19.1)	1.016 (0.809; 1.276)	55.0 (27.4;72.2)	0.450 (0.278; 0.726)
≥80	−242.5 (−62.2; −623.4)	3.425 (1.622; 7.234)	97.8 (90.9; 99.5)	0.022 (0.005; 0.091)	−143.8 (−975.7; 44.8)	2.438 (0.552; 10.757)	52.9 (−9.9; 79.8)	0.471 (0.202; 1.099)
Sex								
Male	65.8 (56.0; 73.4)	0.342 (0.266; 0.440)	93.6 (88.7; 96.4)	0.064 (0.036; 0.113)	23.4 (10.5; 34.4)	0.766 (0.656; 0.895)	50.6 (39.4; 59.8)	0.458 (0.370; 0.566)
Female	78.5 (70.8; 84.2)	0.215 (0.158; 0.292)	100.0 (-) ^a^	0 (-) ^a^	33.4 (22.9; 42.5)	0.666 (0.575; 0.771)	74.3 (62.2; 82.5)	0.257 (0.175; 0.378)
**Symptom**								
Symptomatic	70.0 (57.7; 78.7)	0.300 (0.213; 0.423)	93.2 (83.5; 97.2)	0.068 (0.028; 0.165)	20.6 (3.8; 34.5)	0.794 (0.655; 0.962)	64.5 (51.3; 74.0)	0.355 (0.260; 0.487)
Asymptomatic	68.0 (59.3; 74.8)	0.320 (0.252; 0.407)	94.9 (89.2; 97.6)	0.051 (0.024; 0.108)	8.9 (−4.2; 20.3)	0.911 (0.797; 1.042)	49.8 (36.5; 60.3)	0.502 (0.397; 0.635)
Total								
	72.3 (66.4; 77.2)	0.277 (0.228; 0.336)	95.0 (91.2; 97.2)	0.050 (0.028; 0.088)	29.0 (21.1; 36.2)	0.710 (0.638; 0.789)	61.1 (53.2; 67.6)	0.389 (0.324; 0.468)

^a^ Confidence interval could not be estimated because there were no events among those who were vaccinated, April to June and July refer to pre-delta-dominant and delta-dominant periods, respectively; Symptomatic and asymptomatic were defined as per World Health Organization (WHO) definitions.

## Supplementary Materials

The following supporting information can be downloaded at: https://www.mdpi.com/article/10.3390/ijerph192416958/s1, Table S1: STROBE checklist for case-control studies; Table S2: Effectiveness of COVID-19 vaccines against SARS-CoV-2 infection according to age, sex, and symptom, the 1:1 post-matching study population (April to July); Table S3: Extended districts characteristics of the study population according to SARS-CoV-2 infection status detected by RT-PCR and COVID-19 vaccination status; Table S4: Reduction of COVID-19 vaccines effectiveness against SARS-CoV-2 infection according to age, sex, and symptom; Table S5: Distribution of full-vaccination timing according to age; Table S6: Vaccine effectiveness in the sex and present of symptoms with an adjustment for temporality in incidence of infection.
