# Supplementary material for: Reduction in COVID-19 Vaccine Effectiveness against SARS-CoV-2 Variants in Seoul according to Age, Sex, and Symptoms: A Test-Negative Case-Control Study
Source: Int J Environ Res Public Health. 2022 Dec 16;19(24):16958. doi: 10.3390/ijerph192416958 (PMC9779328; doi:10.3390/ijerph192416958)
Supplement: Supplementary file 1 [file ijerph-19-16958-s001.zip › ijerph-2078832-supplementary.pdf]

Supplementary Table S1. STROBE checklist for case-control studies

	Item No.	Recommendation	Page No.
**Title and abstract**	1	(*a*) Indicate the study's design with a commonly used term in the title or the abstract	1
		(*b*) Provide in the abstract an informative and balanced summary of what was done and what was found	1
**Introduction**			
Background/rationale	2	Explain the scientific background and rationale for the investigation being reported	1-2
Objectives	3	State specific objectives, including any prespecified hypotheses	2
**Methods**			
Study design	4	Present key elements of study design early in the paper	2-3
Setting	5	Describe the setting, locations, and relevant dates, including periods of recruitment, exposure, follow-up, and data collection	2-5
Participants	6	Give the eligibility criteria, and the sources and methods of case ascertainment and control selection. Give the rationale for the choice of cases and controls	4
		For matched studies, give matching criteria and the number of controls per case	3-4 & Figure 1
Variables	7	Clearly define all outcomes, exposures, predictors, potential confounders, and effect modifiers. Give diagnostic criteria, if applicable	4
Data sources/ measurement	8	For each variable of interest, give sources of data and details of methods of assessment (measurement). Describe comparability of assessment methods if there is more than one group	4-5
Bias	9	Describe any efforts to address potential sources of bias	2
Study size	10	Explain how the study size was arrived at	2-3 & Table 1 & Figure 1 & Supplementary Table S3-5
Quantitative variables	11	Explain how quantitative variables were handled in the analyses. If applicable, describe which groupings were chosen and why	2-5
Statistical methods	12	(*a*) Describe all statistical methods, including those used to control for confounding	4-5
		(*b*) Describe any methods used to examine subgroups and interactions	4-5
		(*c*) Explain how missing data were addressed	4-6
		If applicable, explain how matching of cases and controls was addressed	4
		(*e*) Describe any sensitivity analyses	NA
**Results**			
Participants	13	(a) Report numbers of individuals at each stage of study—eg numbers potentially eligible, examined for eligibility, confirmed eligible, included in the study, completing follow-up, and analysed	5

		(b) Give reasons for non-participation at each stage	5
		(c) Consider use of a flow diagram	2 & Figure 1
Descriptive data	14	(a) Give characteristics of study participants (eg demographic, clinical, social) and information on exposures and potential confounders	5-7 & Table 1 & Supplementary Table S3-5
		(b) Indicate number of participants with missing data for each variable of interest	7 & Table 1
Outcome data	15	Report numbers in each exposure category, or summary measures of exposure	5-11
Main results	16	(*a*) Give unadjusted estimates and, if applicable, confounder-adjusted estimates and their precision (eg, 95% confidence interval). Make clear which confounders were adjusted for and why they were included	Supplementary Table S6
		(*b*) Report category boundaries when continuous variables were categorized	2-3, Table 1 & Figure 1
		(*c*) If relevant, consider translating estimates of relative risk into absolute risk for a meaningful time period	NA
Other analyses	17	Report other analyses done—eg analyses of subgroups and interactions, and sensitivity analyses	5-11 & Figure 2-3 & Table 2
**Discussion**			
Key results	18	Summarise key results with reference to study objectives	11-12
Limitations	19	Discuss limitations of the study, taking into account sources of potential bias or imprecision. Discuss both direction and magnitude of any potential bias	13-14
Interpretation	20	Give a cautious overall interpretation of results considering objectives, limitations, multiplicity of analyses, results from similar studies, and other relevant evidence	12-13
Generalisability	21	Discuss the generalisability (external validity) of the study results	14
**Other information**			
Funding	22	Give the source of funding and the role of the funders for the present study and, if applicable, for the original study on which the present article is based	14

Supplementary Table S2. Effectiveness of COVID-19 vaccines against SARS-CoV-2 infection according to age, sex, and symptom, the 1:1 post-matching study population (April to July)

	**April to July Effectiveness, % (Odds ratio value)**			
	Partially vaccinated		Fully vaccinated	
	VE(%)	Odds ratio(95% CI)	VE(%)	Odds ratio(95% CI)
Age group, years (%)				
20-39	71.7 (61.5;79.2)	0.283 (0.208;0.385)	79.3 (73.6;83.8)	0.207 (0.162;0.264)
40-59	68.4 (61.4;74.2)	0.316 (0.258;0.386)	82.9 (74.9;88.3)	0.171 (0.117;0.251)
60-79	67.2 (61.9;71.7)	0.328 (0.283;0.381)	87.8 (81.1;92.1)	0.122 (0.079;0.189)
≥80	-470.5 (-188.3;-1028.9)	5.705 (2.883;11.289)	87.3 (77.0;93.0)	0.127 (0.070;0.230)
Sex				
Male	40.9 (32.7;48.0)	0.591 (0.520;0.673)	75.6 (70.4;80.0)	0.244 (0.200;0.296)
Female	56.6 (50.7;61.8)	0.434 (0.382;0.493)	87.6 (81.9;91.5)	0.124 (0.085;0.181)
Symptom				
Symptomatic	38.1 (27.2;47.4)	0.619 (0.526;0.728)	77.9 (70.5;83.4)	0.221 (0.166;0.295)
Asymptomatic	39.9 (32.7;46.4)	0.601 (0.536;0.673)	76.0 (70.0;80.7)	0.240 (0.193;0.300)
Total				
	49.9 (45.2;54.2)	0.501 (0.458;0.548)	79.7 (75.8;82.9)	0.203 (0.171;0.242)

Supplementary Table S3. Extended districts characteristics of the study population according to SARS-CoV-2 infection status detected by RT-PCR and COVID-19 vaccination status

Characteristics	SARS-CoV-2 infection				Vaccination					
	Controls		Cases		Unvaccinated		Partially vaccinated		Fully vaccinated	
	April to June	July	April to June	July	April to June	July	April to June	July	April to June	July
Total population	35,780	47,060	3,578	4,706	33,696	43,085	3,549	5,669	2,113	3,012
Districts of Seoul										
Center Area	910	1,440	91	144	849	1,309	116	186	36	89
Jongno	595 (1.7)	908 (1.9)	60 (1.7)	93 (2.0)	558 (1.7)	835 (1.9)	75 (2.1)	116 (2.0)	22 (1.0)	50 (1.7)
Junggu	203 (0.6)	337 (0.7)	19 (0.5)	41 (0.9)	184 (0.5)	313 (0.7)	29 (0.8)	46 (0.8)	9 (0.4)	19 (0.6)
Yongsan	112 (0.3)	195 (0.4)	12 (0.3)	10 (0.2)	107 (0.3)	161 (0.4)	12 (0.3)	24 (0.4)	5 (0.2)	20 (0.7)
Northeast Area	8,540	10,610	854	1,061	7,895	9,644	944	1,298	555	729
Dongdaemun	465 (1.3)	551 (1.2)	24 (0.7)	14 (0.3)	405 (1.2)	452 (1.0)	52 (1.5)	74 (1.3)	32 (1.5)	39 (1.3)
Seongdong	2,542 (7.1)	3,176 (6.7)	326 (9.2)	282 (6.0)	2,363 (6.9)	2,845 (6.6)	293 (8.3)	387 (6.8)	212 (10.0)	226 (7.5)
Gwangjin	1,286 (3.6)	1,573 (3.3)	55 (1.5)	119 (2.5)	1,158 (3.4)	1,414 (3.3)	112 (3.2)	176 (3.1)	71 (3.4)	102 (3.4)
Jungnang	1,482 (4.1)	1,784 (3.8)	61 (1.7)	223 (4.7)	1,270 (3.8)	1,642 (3.8)	181 (5.1)	237 (4.2)	92 (4.4)	128 (4.1)
Seongbuk	294 (0.8)	403 (0.9)	15 (0.4)	13 (0.3)	254 (0.8)	315 (0.7)	38 (1.1)	51 (0.9)	17 (0.8)	50 (1.7)
Gangbuk	150 (0.4)	214 (0.5)	10 (0.3)	11 (0.2)	130 (0.4)	186 (0.4)	20 (0.6)	25 (0.4)	10 (0.5)	14 (0.5)
Dobong	315 (0.9)	374 (0.8)	19 (0.5)	35 (0.7)	276 (0.8)	312 (0.7)	43 (1.2)	68 (1.2)	15 (0.7)	29 (1.0)
Nowon	2,006 (5.6)	2,535 (5.4)	344 (9.6)	364 (7.7)	2,039 (6.0)	2,478 (5.8)	205 (5.8)	280 (4.9)	106 (5.0)	141 (4.6)
Northwest Area	640	3,380	64	338	604	3,209	62	347	38	162
Eunpyeong	146 (0.4)	598 (1.3)	10 (0.3)	16 (0.3)	131 (0.4)	500 (1.2)	16 (0.5)	81 (1.5)	9 (0.4)	33 (1.1)
Seodaemun	165 (0.5)	941 (2.0)	23 (0.6)	144 (3.1)	157 (0.5)	935 (2.2)	20 (0.6)	103 (1.8)	11 (0.5)	47 (1.6)
Mapo	329 (0.9)	1,841 (3.9)	31 (0.9)	178 (3.8)	316 (0.9)	1,774 (4.1)	26 (0.7)	163 (2.9)	18 (0.9)	82 (2.7)
Southeast Area	18,210	15,900	1,821	1,590	17,380	14,941	1,674	1,606	977	943
Seocho	981 (2.7)	881 (1.9)	81 (2.3)	81 (1.7)	933 (2.8)	830 (1.9)	82 (2.3)	73 (1.3)	47 (2.2)	59 (2.0)
Gangnam	7,374 (20.6)	6,548 (13.9)	1,017 (28.4)	708 (15.0)	7,400 (22.0)	6,386 (14.8)	614 (17.3)	527 (9.3)	377 (17.8)	343 (11.4)
Songpa	5,668 (15.8)	4,840 (10.3)	366 (10.2)	511 (10.9)	5,183 (15.4)	4,506 (10.5)	541 (15.2)	550 (9.7)	310 (14.7)	295 (9.8)
Gangdong	4,187 (11.7)	3,631 (7.7)	357 (10.0)	290 (6.2)	3,864 (11.5)	3,219 (7.5)	437 (12.3)	456 (8.1)	243 (11.5)	246 (8.2)
Southwest Area	7,480	15,730	748	1,573	6,968	13,982	753	2,232	507	1,089

Yangcheon	202	(0.6)	395	(0.8)	16	(0.4)	15	(0.3)	187	(0.6)	330	(0.8)	20	(0.6)	58	(1.0)	11	(0.5)	22	(0.7)
Gangseo	253	(0.7)	500	(1.1)	16	(0.4)	24	(0.5)	228	(0.7)	405	(0.9)	26	(0.7)	76	(1.4)	15	(0.7)	43	(1.4)
Guro	221	(0.6)	405	(0.9)	13	(0.4)	25	(0.5)	203	(0.6)	347	(0.8)	20	(0.6)	49	(0.9)	11	(0.5)	34	(1.1)
Geumcheon	203	(0.6)	383	(0.8)	10	(0.3)	50	(1.1)	191	(0.6)	368	(0.9)	14	(0.4)	50	(0.9)	8	(0.4)	15	(0.5)
Yeongdeungpo	1,748	(4.9)	3,881	(8.2)	234	(6.5)	302	(6.5)	1,695	(5.0)	3,483	(8.1)	162	(4.6)	453	(8.0)	125	(5.9)	247	(8.2)
Dongjak	2,038	(5.7)	4,322	(9.2)	170	(4.8)	535	(11.4)	1,845	(5.5)	3,891	(9.0)	229	(6.5)	659	(11.6)	134	(6.3)	307	(10.2)
Gwanak	2,815	(7.9)	5,844	(12.4)	289	(8.1)	622	(13.2)	2,619	(7.7)	5,158	(12.0)	282	(7.9)	887	(15.6)	203	(9.6)	421	(14.0)

Supplementary Table S4. Reduction of COVID-19 vaccines effectiveness against SARS-CoV-2 infection according to age, sex, and symptom

**Characteristics**	**April to June**						**July**					
	Controls			Cases			Controls			Cases		
	Un-vaccinated	Partially vaccinated	Fully vaccinated	Un-vaccinated	Partially vaccinated	Fully vaccinated	Un-vaccinated	Partially vaccinated	Fully vaccinated	Un-vaccinated	Partially vaccinated	Fully vaccinated
	30,239	3,440	2,101	3,457	109	12	38,902	5,267	2,891	4,183	402	121
Age group, years (%)												
0-19	3,450	0	0	345	0	0	6,995	4	1	700	0	0
20-39	14,399	384	1,067	1,556	21	8	17,743	749	1,478	1,922	16	59
40-59	10,985	958	367	1,207	23	1	13,039	1,602	759	1,440	74	26
60-79	1,268	2,078	294	315	48	1	1,060	2,904	446	111	309	21
≥80	137	20	373	34	17	2	65	8	207	10	3	15
Sex												
Male	14,680	1,649	1,611	1,716	66	12	19,549	2,303	1,918	2,094	189	94
Female	15,559	1,791	490	1,741	43	0	19,353	2,964	973	2,089	213	27
Symptom												
Symptomatic	3,876	309	180	1,588	38	5	5,264	438	304	2,241	148	46
Asymptomatic	26,363	3,131	1,921	1,869	71	7	33,638	4,829	2,587	1,942	254	75

Supplementary Table S5. Distribution of full-vaccination timing according to age

	April to June										July									
	20-39		40-59		60-79		≥80		Total		20-39		40-59		60-79		≥80		Total	
Total population	17,435		13,541		4,004		583		35,563		21,967		16,940		4,851		308		44,066	
Non-full vaccination	16,345	(93.7)	13,167	(97.2)	3,699	(92.4)	193	(33.1)	33,404	(93.9)	20,224	(92.1)	16,005	(94.5)	4,263	(87.9)	84	(27.3)	40,576	(92.1)
Full-vaccination	1,090	(6.3)	374	(2.8)	305	(7.6)	390	(66.9)	2,159	(6.1)	1,743	(7.9)	935	(5.5)	588	(12.1)	224	(72.7)	3,490	(7.9)
Vaccination time																				
April	-	-	4	(1.1)	15	(4.9)	29	(7.4)	48	(2.2)	2	(0.1)	4	(0.4)	15	(2.6)	16	(7.1)	37	(1.1)
April 01-10	-	-	-	-	-	-	-	-	-	-	-	-	-	-	-	-	-	-	-	-
April 11-20	-	-	-	-	-	-	-	-	-	-	-	-	-	-	-	-	-	-	-	-
April 21-30	-	-	4	(1.1)	15	(4.9)	29	(7.4)	48	(2.2)	2	(0.1)	4	(0.4)	15	(2.6)	16	(7.1)	37	(1.1)
May	20	(1.8)	29	(7.8)	56	(18.4)	210	(53.8)	315	(14.6)	15	(0.9)	30	(3.2)	86	(14.6)	114	(50.9)	245	(7.0)
May 01-10	8	(0.7)	14	(3.8)	13	(4.3)	49	(12.6)	84	(3.9)	5	(0.3)	9	(1.0)	29	(4.9)	25	(11.2)	68	(1.9)
May 11-20	7	(0.6)	8	(2.1)	23	(7.5)	102	(26.1)	140	(6.5)	5	(0.3)	12	(1.2)	30	(5.1)	53	(23.7)	100	(2.9)
May 21-31	5	(0.5)	7	(1.9)	20	(6.6)	59	(15.1)	91	(4.2)	5	(0.3)	9	(1.0)	27	(4.6)	36	(16.1)	77	(2.2)
June	1,070	(98.2)	341	(91.2)	234	(76.7)	151	(38.7)	1,796	(83.2)	1,100	(63.1)	408	(43.6)	237	(40.3)	74	(33.0)	1,819	(52.1)
June 01-10	257	(23.6)	55	(14.7)	14	(4.6)	29	(7.4)	355	(16.4)	234	(13.4)	62	(6.6)	10	(1.7)	17	(7.6)	323	(9.3)
June 11-20	798	(73.2)	261	(69.8)	83	(27.2)	65	(16.7)	1,207	(55.9)	843	(48.4)	296	(31.7)	92	(15.6)	19	(8.5)	1,250	(35.8)
June 21-30	15	(1.4)	25	(6.7)	137	(44.9)	57	(14.6)	234	(10.9)	23	(1.3)	50	(5.3)	135	(23.0)	38	(16.9)	246	(7.0)
July											626	(35.9)	493	(52.7)	250	(42.5)	20	(8.9)	1,389	(39.8)
July 01-10	-	-	-	-	-	-	-	-	-	-	176	(10.1)	120	(12.8)	85	(14.5)	15	(6.7)	396	(11.3)

July 11-20	-	-	-	-	-	-	-	-	-	-	270	(15.5)	275	(29.4)	66	(11.2)	5	(2.2)	616	(17.7)
July 21-31	-	-	-	-	-	-	-	-	-	-	180	(10.3)	98	(10.5)	99	(16.8)	-	-	377	(10.8)

Supplementary Table S6. Vaccine effectiveness in the sex and present of symptoms with an adjustment for temporality in incidence of infection

Adjusted odds ratio (95% CI)	April to June Effectiveness (OR value)								July Effectiveness (OR value)							
	Partially vaccinated				Fully vaccinated				Partially vaccinated				Fully vaccinated			
	OR	%95 CI		*P*	OR	%95 CI		*P*	OR	%95 CI		*P*	OR	%95 CI		*P*
		Lower	Upper			Lower	Upper			Lower	Upper			Lower	Upper	
**Age**																
20-39	1.000	(Reference Group)			1.000	(Reference Group)			1.000	(Reference Group)			1.000	(Reference Group)		
40-59	0.432	0.230	0.809	0.009	0.275	0.034	2.231	0.227	-	-	-	-	-	-	-	-
60-79	0.395	0.228	0.683	0.001	0.568	0.069	4.640	0.597	-	-	-	-	-	-	-	-
≥80	12.107	4.877	30.053	<0.001	0.573	0.120	2.748	0.487	-	-	-	-	-	-	-	-
**Sex**																
Male	1.000	(Reference Group)			1.000	(Reference Group)			1.000	(Reference Group)			1.000	(Reference Group)		
Female (1)	0.687	0.460	1.026	0.067	-	-	-	-	1.057	0.857	1.306	0.603	0.608	0.390	0.948	0.028
**Symptom**																
Symptomatic	1.000	(Reference Group)			1.000	(Reference Group)			1.000	(Reference Group)			1.000	(Reference Group)		
Asymptomatic (1)	0.194	0.125	0.301	<0.001	0.138	0.042	0.454	0.001	0.127	0.098	0.163	<0.001	0.169	0.112	0.256	<0.001
